# Ammonium benzene­phospho­nate

**DOI:** 10.1107/S1600536808023143

**Published:** 2008-07-26

**Authors:** Zhen Lin, Xiu-Qing Lei, Sheng-Di Bai, Seik Weng Ng

**Affiliations:** a Shanxi Vocational and Technical College of Biological Applications, Taiyuan 030031, Shanxi, People’s Republic of China; b Institute of Applied Chemistry, Shanxi University, Taiyuan 030006, People’s Republic of China; c Department of Chemistry, University of Malaya, 50603 Kuala Lumpur, Malaysia

## Abstract

In the crystal structure of the title salt, NH_4_^+^.[(C_6_H_5_)P(O)_2_(OH)]^−^ or NH_4_^+^·C_6_H_6_O_3_P^−^, the N and O atoms inter­act *via* hydrogen bonds to generate a layer motif. The phenyl rings are stacked above and below this layer, sandwiching the hydrogen-bonded layer.

## Related literature

For the crystal structure of benzene­phospho­nic acid, see: Weakley (1976 ▶); Mahmoudkhani & Langer (2002 ▶). For the crystal structure of the 1:1 co-crystal of ammonium benzene­phospho­nate and benzene­phospho­nic acid, see: Rao & Vidyasagar (2005 ▶).
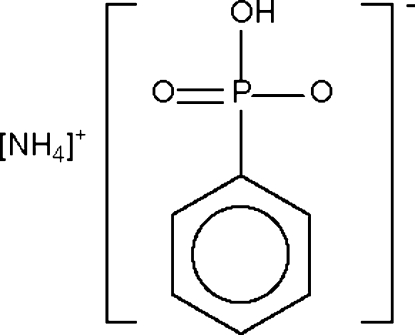


## Experimental

### 

#### Crystal data

NH_4_^+^·C_6_H_6_O_3_P^−^
*M* *_r_* = 175.12
Orthorhombic, 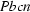

*a* = 31.122 (2) Å
*b* = 7.1249 (5) Å
*c* = 7.9441 (5) Å
*V* = 1761.5 (2) Å^3^
*Z* = 8
Mo *K*α radiation
μ = 0.27 mm^−1^
*T* = 293 (2) K
0.4 × 0.4 × 0.2 mm

#### Data collection

Bruker SMART 1000 diffractometer
Absorption correction: multi-scan (*SADABS*; Sheldrick, 1996 ▶) *T*_min_ = 0.807, *T*_max_ = 0.947
7880 measured reflections
1565 independent reflections
1540 reflections with *I* > 2σ(*I*)
*R*_int_ = 0.027

#### Refinement

*R*[*F* ^2^ > 2σ(*F* ^2^)] = 0.066
*wR*(*F* ^2^) = 0.173
*S* = 1.27
1565 reflections
120 parameters
11 restraints
H atoms treated by a mixture of independent and constrained refinement
Δρ_max_ = 0.31 e Å^−3^
Δρ_min_ = −0.39 e Å^−3^

### 

Data collection: *SMART* (Bruker, 2000 ▶); cell refinement: *SAINT* (Bruker, 2000 ▶); data reduction: *SAINT*; program(s) used to solve structure: *SHELXS97* (Sheldrick, 2008 ▶); program(s) used to refine structure: *SHELXL97* (Sheldrick, 2008 ▶); molecular graphics: *X-SEED* (Barbour, 2001 ▶); software used to prepare material for publication: *publCIF* (Westrip, 2008 ▶).

## Supplementary Material

Crystal structure: contains datablocks I, global. DOI: 10.1107/S1600536808023143/xu2441sup1.cif

Structure factors: contains datablocks I. DOI: 10.1107/S1600536808023143/xu2441Isup2.hkl

Additional supplementary materials:  crystallographic information; 3D view; checkCIF report

## Figures and Tables

**Table 1:** Hydrogen-bond geometry (Å, °)

*D*—H⋯*A*	*D*—H	H⋯*A*	*D*⋯*A*	*D*—H⋯*A*
O1—H1⋯O3^i^	0.85 (1)	1.71 (2)	2.526 (3)	163 (4)
N1—H11⋯O2	0.85 (1)	1.91 (1)	2.762 (4)	175 (3)
N1—H12⋯O3^ii^	0.85 (1)	1.99 (1)	2.814 (4)	164 (3)
N1—H13⋯O1^iii^	0.85 (1)	2.09 (2)	2.940 (4)	173 (3)
N1—H14⋯O2^iv^	0.85 (1)	1.93 (1)	2.775 (4)	177 (3)

Symmetry codes: (i) 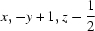; (ii) 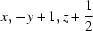; (iii) 
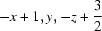
; (iv) 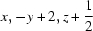.

**Fig. 1.**
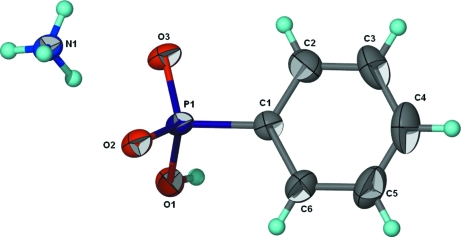
The molecular structure, showing the atom-numbering scheme. Displacement ellipsoids were drawn at the 30% probability level.

## supplementary crystallographic information

### Comment

The title compound (Scheme I, Fig. 1) is a side-product in the synthesis of 2-methylphenylamidinium phenylphosphinate.

### Experimental

*m*-Tolunitrile (0.6 ml, 5 mmol) and lithium bis(trimethylsilyl)amide (0.83 g, 5 mmol) were dissolved in THF (30 ml) at 273 K. The yellow solution was cooled to 195 K. Dichlorophenylphosphine (0.7 ml, 5 mmol) was added. The solution was kept at this temperature for an hour before being allowed to react at room temperature overnight. The solvent was removed and the residue extracted with dichloromethane to give a light yellow oil. The oil was dissolved in acetonitrile (30 ml) and 30% hydrogen peroxide (0.56 cm l, 5 mmol) was added. After 24 h, the solution was filtered. Colorless crystals of 2-methylphenylamidinium phenylphosphinate were first obtained; the second crop yielded the title compound (yield 0.04 g).

### Refinement

The hydroxy and ammonium H atoms were located in a difference Fourier map, and were refined with distance restraints of O—H = N—H 0.85 (1) Å and H···H 1.39 (1) Å. Their temperature factors were freely refined. The aromatic H atoms were placed at calculated positions, and were included in the refinement in the riding model approximation, with *U*_iso_(H) = 1.2*U*_eq_(C).

### Crystal data

**Table:**

NH_4_^+^·C_6_H_6_O_3_P^–^	*F*_000_ = 736
*M**_r_* = 175.12	*D*_x_ = 1.321 Mg m^−^^3^
Orthorhombic, *P**b**c**n*	Mo *K*α radiation λ = 0.71073 Å
Hall symbol: -P 2n 2ab	Cell parameters from 3647 reflections
*a* = 31.122 (2) Å	θ = 2.6–27.5º
*b* = 7.1249 (5) Å	µ = 0.27 mm^−^^1^
*c* = 7.9441 (5) Å	*T* = 293 (2) K
*V* = 1761.5 (2) Å^3^	Block, colorless
*Z* = 8	0.4 × 0.4 × 0.2 mm

### Data collection

**Table:**

Bruker SMART 1000 diffractometer	1565 independent reflections
Radiation source: fine-focus sealed tube	1540 reflections with *I* > 2σ(*I*)
Monochromator: graphite	*R*_int_ = 0.027
*T* = 293(2) K	θ_max_ = 25.0º
φ and ω scans	θ_min_ = 1.3º
Absorption correction: multi-scan(SADABS; Sheldrick, 1996)	*h* = −37→30
*T*_min_ = 0.807, *T*_max_ = 0.947	*k* = −8→6
7880 measured reflections	*l* = −9→9

### Refinement

**Table:**

Refinement on *F*^2^	Secondary atom site location: difference Fourier map
Least-squares matrix: full	Hydrogen site location: inferred from neighbouring sites
*R*[*F*^2^ > 2σ(*F*^2^)] = 0.066	H atoms treated by a mixture of independent and constrained refinement
*wR*(*F*^2^) = 0.173	*w* = 1/[σ^2^(*F*_o_^2^) + (0.073*P*)^2^ + 2.2855*P*] where *P* = (*F*_o_^2^ + 2*F*_c_^2^)/3
*S* = 1.27	(Δ/σ)_max_ = 0.001
1565 reflections	Δρ_max_ = 0.31 e Å^−^^3^
120 parameters	Δρ_min_ = −0.39 e Å^−^^3^
11 restraints	Extinction correction: none
Primary atom site location: structure-invariant direct methods	

### Fractional atomic coordinates and isotropic or equivalent isotropic displacement parameters (Å^2^)

**Table:**

	*x*	*y*	*z*	*U*_iso_*/*U*_eq_	
P1	0.42250 (3)	0.69330 (12)	0.59684 (10)	0.0317 (3)	
O1	0.45014 (8)	0.6911 (4)	0.4300 (3)	0.0410 (7)	
H1	0.4413 (14)	0.607 (4)	0.363 (4)	0.056 (13)*	
O2	0.43772 (8)	0.8608 (3)	0.6936 (3)	0.0403 (6)	
O3	0.42584 (8)	0.5088 (3)	0.6853 (3)	0.0431 (7)	
N1	0.46015 (10)	0.8111 (4)	1.0270 (4)	0.0373 (7)	
H11	0.4547 (9)	0.827 (4)	0.9228 (14)	0.053 (13)*	
H12	0.4455 (8)	0.720 (3)	1.065 (3)	0.052 (13)*	
H13	0.4869 (4)	0.786 (4)	1.038 (4)	0.043 (11)*	
H14	0.4541 (9)	0.910 (2)	1.081 (3)	0.048 (12)*	
C1	0.36793 (11)	0.7216 (5)	0.5282 (5)	0.0381 (8)	
C2	0.33569 (14)	0.6096 (7)	0.5945 (6)	0.0566 (11)	
H2	0.3423	0.5194	0.6751	0.068*	
C3	0.29380 (15)	0.6318 (9)	0.5410 (8)	0.0742 (15)	
H3	0.2722	0.5584	0.5879	0.089*	
C4	0.28380 (16)	0.7611 (9)	0.4195 (8)	0.0802 (16)	
H4	0.2556	0.7725	0.3822	0.096*	
C5	0.31517 (17)	0.8734 (8)	0.3528 (7)	0.0755 (15)	
H5	0.3082	0.9619	0.2711	0.091*	
C6	0.35723 (14)	0.8548 (6)	0.4073 (6)	0.0565 (11)	
H6	0.3785	0.9320	0.3627	0.068*	

### Atomic displacement parameters (Å^2^)

**Table:**

	*U*^11^	*U*^22^	*U*^33^	*U*^12^	*U*^13^	*U*^23^
P1	0.0411 (5)	0.0288 (5)	0.0252 (5)	−0.0007 (3)	−0.0013 (3)	0.0022 (3)
O1	0.0427 (14)	0.0516 (16)	0.0289 (13)	−0.0082 (12)	−0.0003 (11)	−0.0042 (11)
O2	0.0564 (15)	0.0319 (12)	0.0326 (13)	−0.0039 (11)	−0.0083 (11)	−0.0017 (10)
O3	0.0605 (16)	0.0326 (13)	0.0362 (13)	0.0034 (12)	−0.0034 (11)	0.0064 (11)
N1	0.0479 (19)	0.0322 (16)	0.0318 (16)	−0.0016 (13)	−0.0031 (14)	0.0005 (13)
C1	0.0419 (19)	0.0355 (18)	0.0369 (19)	0.0011 (15)	0.0009 (15)	−0.0046 (15)
C2	0.054 (2)	0.060 (3)	0.056 (3)	−0.008 (2)	0.001 (2)	0.002 (2)
C3	0.041 (2)	0.092 (4)	0.089 (4)	−0.011 (2)	0.004 (2)	−0.011 (3)
C4	0.045 (3)	0.099 (4)	0.097 (4)	0.016 (3)	−0.018 (3)	−0.019 (4)
C5	0.066 (3)	0.078 (3)	0.082 (3)	0.017 (3)	−0.026 (3)	0.011 (3)
C6	0.055 (3)	0.055 (2)	0.060 (3)	0.005 (2)	−0.011 (2)	0.014 (2)

### Geometric parameters (Å, °)

**Table:**

P1—O3	1.494 (2)	C1—C6	1.391 (5)
P1—O2	1.497 (2)	C2—C3	1.380 (7)
P1—O1	1.580 (3)	C2—H2	0.9300
P1—C1	1.795 (4)	C3—C4	1.370 (8)
O1—H1	0.85 (1)	C3—H3	0.9300
N1—H11	0.85 (1)	C4—C5	1.369 (8)
N1—H12	0.85 (1)	C4—H4	0.9300
N1—H13	0.85 (1)	C5—C6	1.385 (6)
N1—H14	0.85 (1)	C5—H5	0.9300
C1—C2	1.386 (6)	C6—H6	0.9300
			
O3—P1—O2	115.98 (14)	C3—C2—C1	120.1 (5)
O3—P1—O1	110.38 (14)	C3—C2—H2	120.0
O2—P1—O1	105.45 (14)	C1—C2—H2	120.0
O3—P1—C1	107.91 (16)	C4—C3—C2	120.6 (5)
O2—P1—C1	111.44 (16)	C4—C3—H3	119.7
O1—P1—C1	105.14 (15)	C2—C3—H3	119.7
P1—O1—H1	111 (3)	C5—C4—C3	120.3 (5)
H11—N1—H12	109.6 (14)	C5—C4—H4	119.9
H11—N1—H13	108.8 (14)	C3—C4—H4	119.9
H12—N1—H13	109.2 (14)	C4—C5—C6	119.8 (5)
H11—N1—H14	109.7 (14)	C4—C5—H5	120.1
H12—N1—H14	110.1 (14)	C6—C5—H5	120.1
H13—N1—H14	109.5 (14)	C5—C6—C1	120.5 (4)
C2—C1—C6	118.8 (4)	C5—C6—H6	119.8
C2—C1—P1	120.3 (3)	C1—C6—H6	119.8
C6—C1—P1	120.9 (3)		
			
O3—P1—C1—C2	15.9 (4)	P1—C1—C2—C3	−179.9 (4)
O2—P1—C1—C2	−112.6 (3)	C1—C2—C3—C4	1.5 (8)
O1—P1—C1—C2	133.7 (3)	C2—C3—C4—C5	−1.7 (9)
O3—P1—C1—C6	−163.7 (3)	C3—C4—C5—C6	0.6 (9)
O2—P1—C1—C6	67.8 (4)	C4—C5—C6—C1	0.6 (8)
O1—P1—C1—C6	−45.9 (4)	C2—C1—C6—C5	−0.8 (7)
C6—C1—C2—C3	−0.3 (7)	P1—C1—C6—C5	178.8 (4)

### Hydrogen-bond geometry (Å, °)

**Table:**

*D*—H···*A*	*D*—H	H···*A*	*D*···*A*	*D*—H···*A*
O1—H1···O3^i^	0.85 (1)	1.71 (2)	2.526 (3)	163 (4)
N1—H11···O2	0.85 (1)	1.91 (1)	2.762 (4)	175 (3)
N1—H12···O3^ii^	0.85 (1)	1.99 (1)	2.814 (4)	164 (3)
N1—H13···O1^iii^	0.85 (1)	2.09 (2)	2.940 (4)	173 (3)
N1—H14···O2^iv^	0.85 (1)	1.93 (1)	2.775 (4)	177 (3)

Symmetry codes: (i) *x*, −*y*+1, *z*−1/2; (ii) *x*, −*y*+1, *z*+1/2; (iii) −*x*+1, *y*, −*z*+3/2; (iv) *x*, −*y*+2, *z*+1/2.
